# Atlanto-axial subluxation secondary to a neglected odontoid fracture

**DOI:** 10.1259/bjrcr.20220110

**Published:** 2022-11-01

**Authors:** Masatsugu Tsukamoto, Tadatsugu Morimoto, Takaomi Kobayashi, Hirohito Hirata, Tomohito Yoshihara, Masaaki Mawatari

**Affiliations:** 1 Department of Orthopaedic Surgery, Faculty of Medicine, Saga University, Saga, Japan

## Abstract

A 81-year-old female had chronic renal failure and was undergoing hemodialysis, visited orthopaedic clinic after striking her head on the ceiling of a car while driving on a rough road. An odontoid fracture went unidentified on the initial radiograph. One month later, she came to our hospital with persistent neck pain. A radiography and computed tomography revealed a C1–two subluxation secondary to the fracture. Posterior occipito–C1–C2–C3 fixation was performed, and the patient wore a halo-vest for two-month post-surgery. After two months, the halo-vest was removed, and the patient was not experiencing any pain or neurological deficits. In older patients, even minor head trauma can result in cervical vertebral fractures. Therefore, potential fractures should be considered during initial evaluations to avoid the serious consequences of an incorrect initial diagnosis. Care should be taken when choosing between conservative or surgical treatments, considering all potential risks and complications.

## Background

Cervical spine (C-spine) fractures are common in older patients. In recent years, the incidence of these injuries has increased owing to an increased frequency of detection in super-aging societies and the widespread use of advanced diagnostic imaging modalities.^1^ The initial diagnosis of C-spine injury depends on appropriate, evidence-based patient selection as well as the availability and accurate interpretation of radiographic information. Neglected spinal injuries are defined as injuries identified or treated late, when options are limited.^2^ An overlooked diagnosis is the most common cause of delayed presentation. Here, we report a case of C-spine (C2) fracture, in which an initial misdiagnosis resulted in serious clinical consequences.

## Clinical presentation

**Figure 1.**
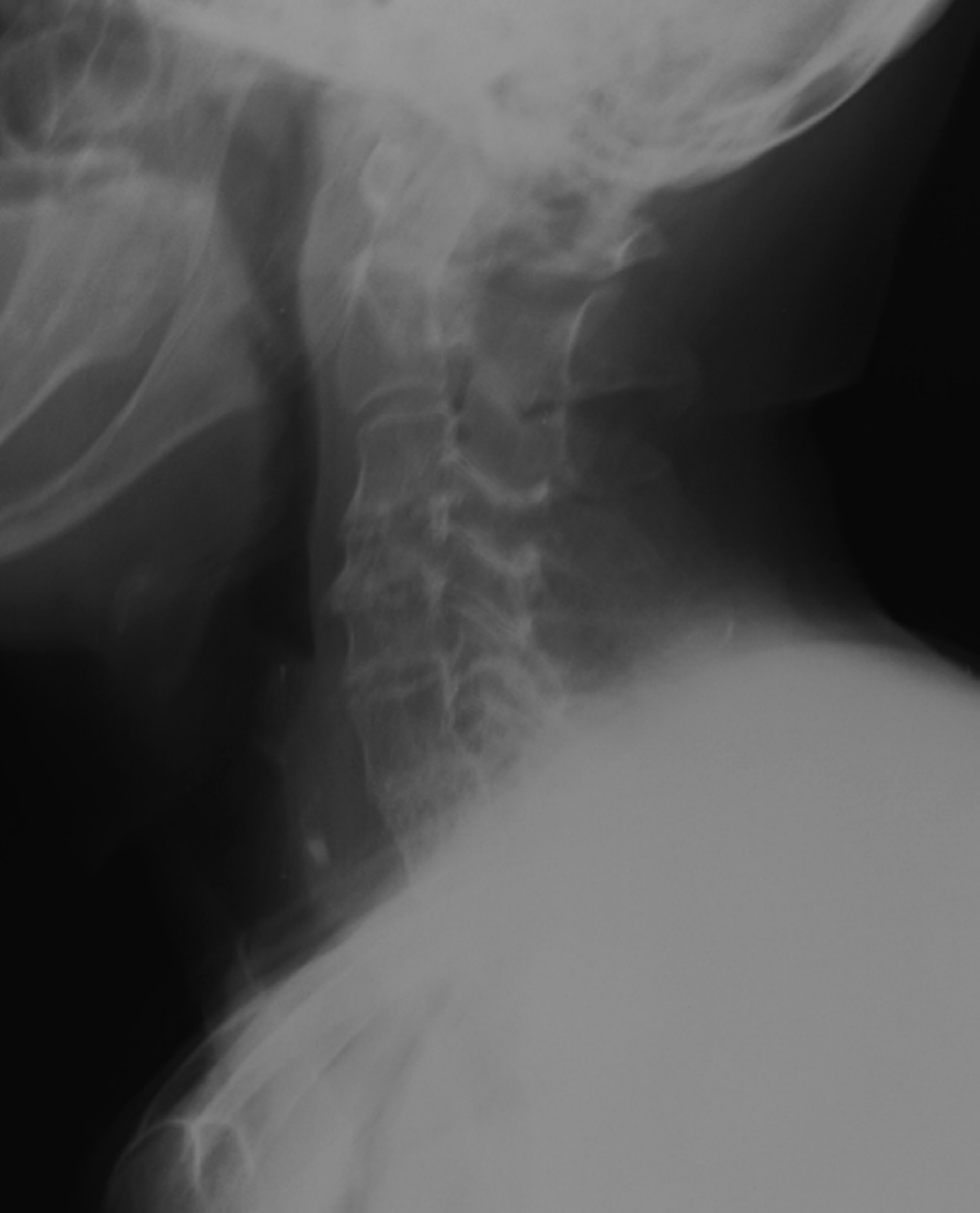
Radiograph of the lateral cervical spine taken during the initial evaluations.

A 81-year-old female presented at our hospital with a chief complaint of subjective neck pain. The patient had previously been involved in a minor vehicular incident. As a passenger in the back seat of a car, the patient hit her head on the ceiling while traveling on a rough road. The patient did not lose consciousness and was able to walk after the injury. The patient visited an orthopedic clinic after experiencing neck pain. C-spine radiography was performed, but no fracture was noted (Figure 1). The neck pain gradually worsened, and the patient eventually presented to our institution. During physical examination, there was no tenderness along the midline of the neck. The patient was able to flex the neck forward, but backward flexion caused severe pain. The patient was able to walk and was not experiencing neurological deficits. Clinically, we suspected a diagnosis of cervical sprain.

**Figure 2.**
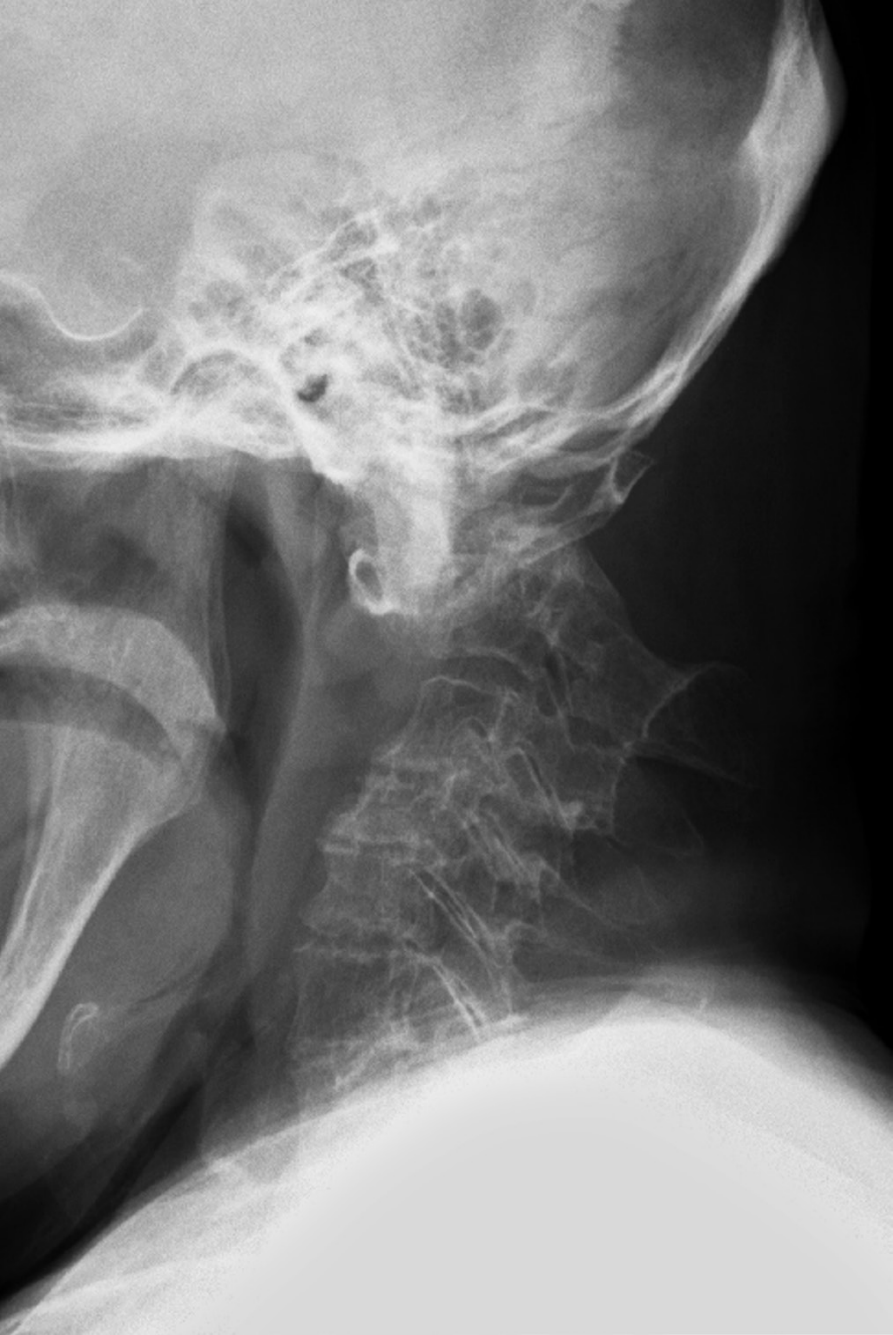
A plain radiograph of the lateral cervical spine taken at our hospital revealed an atlanto-axial (C1–2) dislocation secondary to an odontoid fracture.

**Figure 3.**
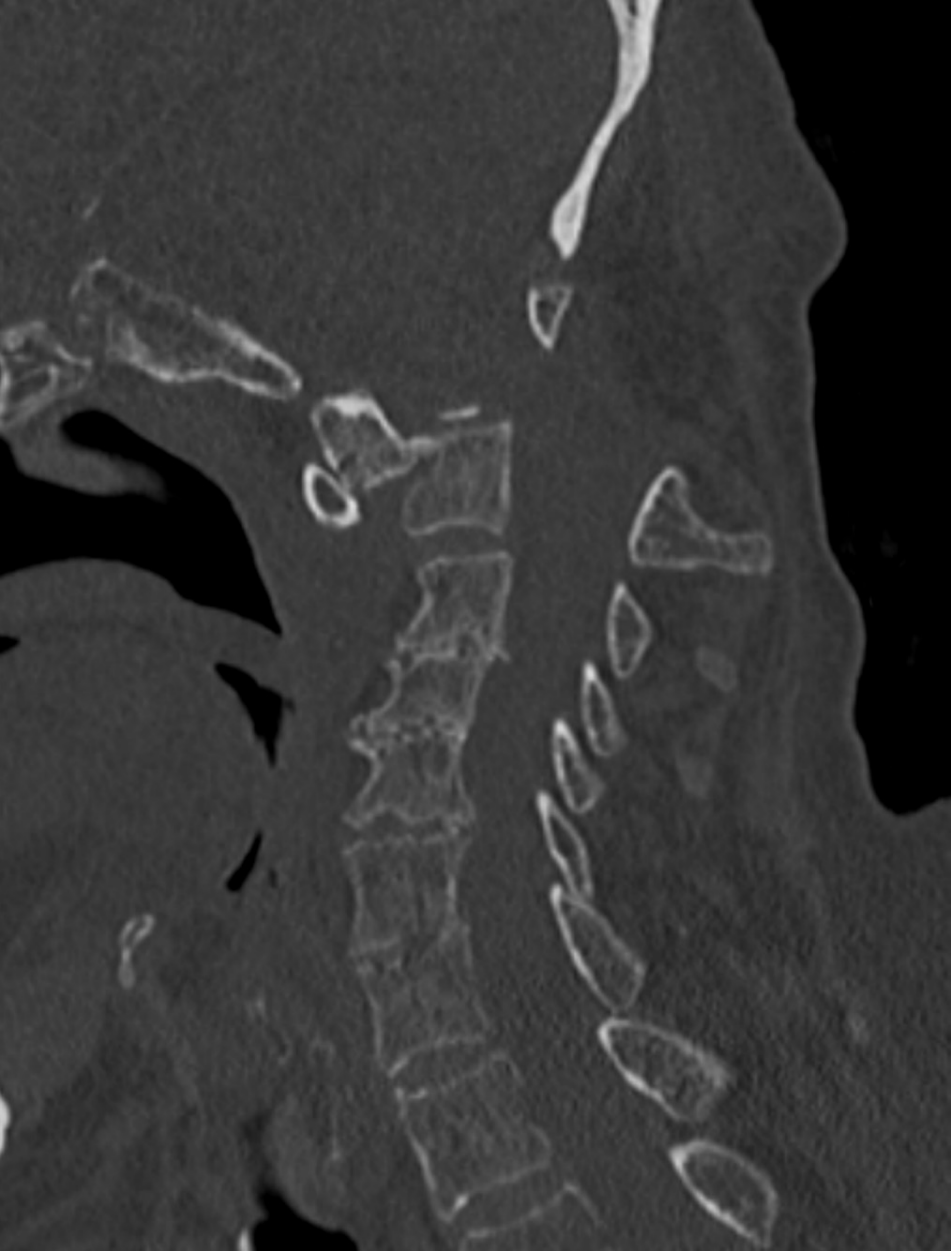
Computed tomography scan of the cervical spine revealed nonunion of an odontoid fracture with C1–two subluxation.

A plain radiograph of the C-spine revealed an atlanto-axial (C1–2) dislocation secondary to odontoid fractures (Figure 2). A computed tomography (CT) scan (Figure 3) revealed nonunion of an odontoid fracture with C1–two subluxation.

## Differential diagnosis

Although initially thought to be a cervical sprain, a plain radiograph of the C-spine one month after the initial trauma revealed a fracture of the cervical vertebral dental process and a subluxation of the annular axial vertebra. Therefore, the clinical diagnosis was an annular-axial subluxation with involvement of a missed axial vertebral fracture.

Examinations of the patient’s medical history as well as clinical, hematological, biochemical, and radiological data did not reveal any findings suspicious of fracture secondary to bone malignancy or of bone degradation associated with infection. The patient was undergoing hemodialysis because of chronic renal failure due to multiple kidney cysts. Therefore, the fracture was thought to be facilitated by osteoporosis.

## Treatment

Our patient did not have progressive myelopathy but was experiencing severe pain and disabilities in activities of daily living. Therefore, we decided to perform surgery. The patient was admitted to the orthopedic department and fitted with a halo-vest. Under fluoroscopic guidance, the halo-vest was used for reduction of the subluxation. From the contrast CT images, we concluded the patient at risk of vertebral artery disruption during reduction of the subluxation. Consequently, the procedure was performed while the patient was awake. Good alignment was attained without a loss of consciousness. Posterior occipito–C1–C2–C3 fixation using a C2 magerl, C2 lamina, and C3 lateral mass screw was performed on the same day. The O–C2 angle was fixed at 15°, which is approximately the same angle enforced by the halo-vest during reduction. The torque for screw insertion had been poor, and there were concerns about bone fragility. Therefore, a hybrid method of internal and external fixation was used, and the patient had to continue wearing the halo-vest post-operatively for two months.

### Outcome and follow-up

**Figure 4.**
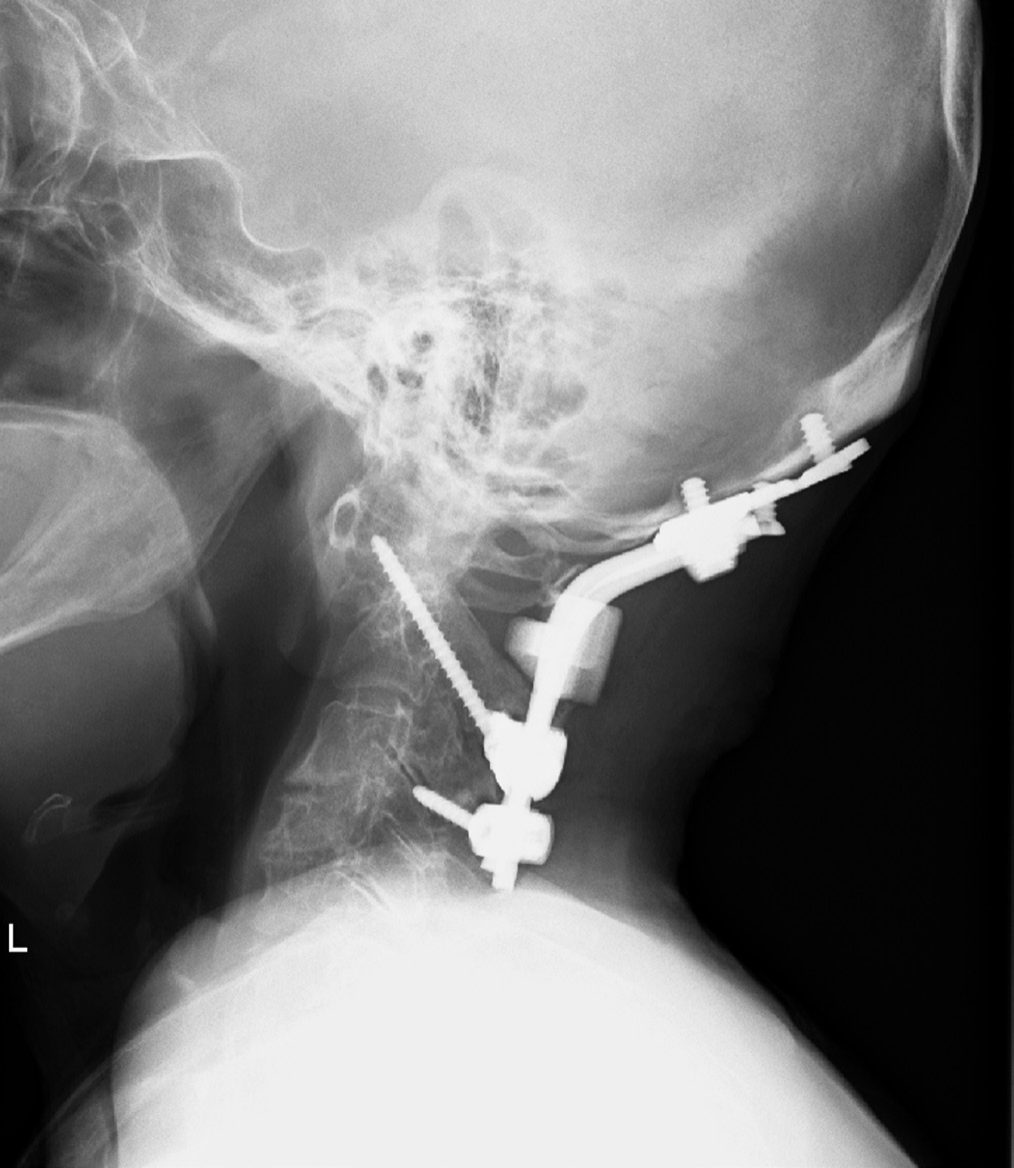
Postoperative plain radiograph of the lateral cervical spine showing fusion of the fracture and no evidence of instrumentation loosening.

Two months after surgery, the halo-vest was removed, and the patient was not experiencing any pain or neurological deficits. The patient was subject to regular follow-up radiography, which revealed fusion of the fracture and confirmed there was no loosening of the fixation instrumentation (Figure 4). The patient succumbed to another disease after 18 months of follow-up.

## Discussion

Odontoid fractures are the most common fractures of the axis and are the most common types of C-spine fractures in patients over 65 years old.^1^ Expansion of the aging population in the United States has coincided with a more-than-doubled incidence of odontoid fractures over the past decade.^1^ Compared with other parts of the skeleton, a lower bone density in the axial vertebrae and consequent susceptibility to osteoporosis contributes to the high incidence of axial vertebral odontoid process fractures in older patients.^3^ In addition, degeneration of the middle and lower cervical vertebrae causes stress to be concentrated in the upper cervical vertebrae. Therefore, the upper C-spine, and especially the axial vertebrae, should be recognized as common sites of fragility fractures in these patients. The likelihood of C-spine fractures in older patients is exacerbated by impaired mobility and an inability to break a fall by holding out their hands. Therefore, after a fall, upper C-spine fractures should be investigated, even in cases of mild trauma indirectly related to the spine (*e.g.,* head contusions). According to a previous meta-analysis, the sensitivities of plain radiography and CT for the identification of C-spine injuries following blunt trauma are 58% (39–76%) and 98%, respectively.^4^ Therefore, in cases of suspected C-spine injury, CT should be used as the primary imaging modality.^5^ However, some fragile bone fractures in older patients cannot be identified using C-spine CT, and in cases of unexplained, persistent pain, MRI should be considered as an alternative. Further, subclinical fractures in other regions of the spine and pelvis should be investigated.^6^

The present case is an example of initial misdiagnosis, in which the possibility of odontoid process fracture was overlooked. Overlooked C-spine injuries are far more common than those occurring in the thoracolumbar spine and sacrum. Upper C-spine injuries in older patients are easily missed and may be diagnosed late, when the dislocation becomes enlarged.^7^ A lack of symptomology, inaccurate findings during initial investigations, width of the spinal canal, and rarity of neuropathy in these cases are factors contributing to a late diagnosis. From a clinical perspective, a late diagnosis may occur because minor trauma (*e.g.,* from a fall) is not expected to cause a fracture, intracranial lesions are more likely to be investigated in cases of head contusion than upper C-spine injuries, and greater attention is paid to the existing degeneration of the middle and lower C-spine during radiography. Therefore, to prevent overlooking a fracture, bidirectional radiographic imaging performed at different times is recommended.

It is well known that type II odontoid fractures frequently go into nonunion; in the literature, the rate of nonunion varies between 26% and 76%.^8^ Undiagnosed odontoid fractures may lead to chronic C1–two instability and progressive myelopathy. There is a paucity of available information aimed at guiding the management of neglected upper C-spine injuries, and there is no standardized method of treatment.^7,9,10^ A more conservative approach is recommended for older patients with stable, non-progressive C1–two deformity.^7^ Preoperative traction is a reasonable means of attempting to correct the spinal deformity before the placement of internal fixation devices. With advances in spinal instrumentation, more reliable occipital–C1–C2 or C1–two fixation methods are available. More aggressive surgical treatment is recommended for patients with progressive myelopathy resulting from dynamic instability.

Neglected upper C-spine injuries are difficult to treat, and the choice of treatment, whether surgical, external, or surgical and external fixation, should be carefully considered on a case-by-case basis. A late diagnosis lessens the likelihood of a satisfactory clinical outcome compared with treatment of the same injury that was correctly diagnosed at initial presentation. Persistent pain and progressive deformity may require surgical stabilization, even if the injury is conservatively treatable. Sometimes, the underlying injury may be fatal despite minor clinical manifestations. In older patients, minor trauma can cause C-spine fractures, and this should be given careful consideration during initial diagnosis.

## Learning points

- In older patients, even minor head trauma may result in cervical vertebral fractures, and this should be carefully considered during the initial response.
- A fracture overlooked during initial evaluations can complicate later treatment responses and have serious consequences for older patients.
- The choice between conservative or surgical treatment for cervical spine fractures in older patients should take all potential risks and complications into account.
- Before surgery, potential neurovascular complications, the angles of repair and fixation, and bone quality (bone fusion) should be carefully considered.
